# Dynamic Immune Response Landscapes of Avian Peripheral Blood Post-Vaccination Against Infectious Bronchitis Virus Infection

**DOI:** 10.3390/vaccines13020146

**Published:** 2025-01-30

**Authors:** Xuefeng Li, Yumeng Liang, Yu Zhang, Zheyi Liu, Lu Cui, Miaomiao Xi, Shufeng Feng, Xiaoxiao Liu, Yongxin Zhu, Shengwang Liu, Hai Li

**Affiliations:** 1 Division of Avian Infectious Diseases, State Key Laboratory of Animal Disease Control and Prevention, National Poultry Laboratory Animal Resource Center, Harbin Veterinary Research Institute, The Chinese Academy of Agricultural Sciences, Harbin 150069, China; lixuefeng57@126.com (X.L.); m1584702@163.com (Y.L.); zhangyu05@caas.cn (Y.Z.); liuzy0701@126.com (Z.L.); cuilu099@163.com (L.C.); 2 School of Basic Medical Sciences, Xi’an Jiaotong University Health Science Center, Xi’an Jiaotong University, Xi’an 710061, China; ximiaom@stu.xjtu.edu.cn (M.X.); feng_xjtu0204@163.com (S.F.); liuxiaoxiao12@stu.xjtu.edu.cn (X.L.); zhuyongxin2023@163.com (Y.Z.)

**Keywords:** avian infectious bronchitis virus, adaptive immune responses, vaccine, immune landscape

## Abstract

**Background/Objectives**: Despite decades of extensive vaccinations against avian infectious bronchitis virus (IBV) infection, outbreaks caused by constantly emerging variants due to genome recombination between different viral strains, including vaccine strains, occur annually worldwide. The development of novel vaccines with favorable safety and effectiveness is required but is hindered by a limited understanding of vaccination against IBV. **Methods**: Here, we performed a comprehensive analysis of the in vivo dynamics of peripheral blood mononuclear cells (PBMCs) in specific pathogen-free chickens inoculated with the widely used live attenuated IBV vaccine strain H120 at single-cell level, using high-throughput single-cell transcriptome sequencing (scRNA-seq). **Results**: High-quality sequencing dataset for four scRNA-seq data containing the transcriptomes of 29,846 individual chicken PBMCs were obtained, defining 22 populations and 7 cell types based on distinct molecular signatures and known markers. Further integrative analysis constructed the time series dynamic cell transition and immune response landscapes within the two weeks post-prime vaccination against IBV. Enhanced crosstalk between antigen-presenting cells and T lymphocytes was revealed as early as four days post-vaccination. The specific immune cell populations and their comprehensive cellular and molecular networks involved in the initiation phase of antiviral adaptive immune responses were elucidated in details. **Conclusions**: Our study provides a comprehensive view of the dynamic initiation of immune responses in chickens against IBV infection at the cellular and molecular levels, which provides theoretical support and potential solutions for the future rational design of safe and effective vaccines, the augmentation of the efficacy of current vaccines, and the optimization of immune programs.

## 1. Introduction

Avian infectious bronchitis (IB) is caused by avian coronaviruses, members of the genus Gamma coronavirus, family *Coronaviridae* and order *Nidovirales*. The prototype of avian coronaviruses is avian infectious bronchitis virus (IBV), the first coronavirus being identified in the 1930’s followed by the mouse hepatitis virus and the transmissible gastroenteritis virus in the next ten years [1,2,3]. Despite being of a different genus, IBV has many similarities with SRAS-CoV-2 in the pathology of the respiratory system and vaccination [4,5]. Thus, as the first identified and most extensively investigated naturally occurring coronavirus, IBV may be considered as a useful model for studying the evolution, transmission, pathogenesis, and prevention of coronavirus.

Vaccination has been extensively performed to control IBV infections for decades. Live attenuated vaccines are widely administered for prime vaccination in layers and breeders and is sufficient to protect broilers. Inactivated vaccines are normally used to boost the immunity against IBV in layers and breeders before the onset of egg production. Meanwhile, outbreaks caused by constantly emerging variants due to genome recombination between different viral strains, including vaccine strains, occur worldwide annually [6,7]. Like other coronaviruses, for example, SRAS-CoV-2, IBV is susceptible to mutation and genome recombination between different viral strains, including vaccine strains, and is therefore highly diverse. The cross-protection among strains is low. However, other types of vaccines, such as inactivated and subunit vaccines, still encounter issues like poor immunogenicity and low efficiency, which are often difficult to overcome with traditional immune-regulating strategies like adjuvants [8,9]. The development of novel vaccines against IBV infection with high efficacy and lower biosafety risks is necessary but is hindered by a limited understanding of the initiation of antiviral adaptive immune responses by IBV vaccination. In fact, chickens have made significant contributions to the development of immunology, particularly in the understanding of adaptive immunity [10,11]. For example, B lymphocytes, which are crucial for the adaptive immune response, were first identified in chickens, because of the unique immune organ of birds, the bursa of Fabricius. Interferon, a protein that plays a key role in the immune response to viruses, was also first described in chicken embryos exposed to the influenza virus in 1957. Compared with mammals, chickens have a compact and simple major histocompatibility complex (MHC), which has been instrumental in studying genetic resistance and susceptibility to infectious diseases. These contributions have been fundamental in advancing our understanding of immunological processes and have had a lasting impact on both veterinary and human medicine. However, the chicken immune system is complex, with unique features such as the bursa of Fabricius and the absence of lymph nodes, which makes it difficult to fully understand the complexity of chicken immune responses according to current advances in human and mammal immune system [12]. This highlights the need for continued research to improve our understanding of the chicken immune system and adaptive immunity.

The initiation of vertebrate adaptive immune responses is a complex process, involving the interaction result of a large number of innate and adaptive immune cells. Single-cell transcriptome sequencing (scRNA-seq) is a powerful technology for comprehensive elucidation of immune system heterogeneity at single-cell resolution. It has been primarily used to investigate the immune systems of humans and model animals [13,14]. Compared to humans and mice, chickens have significant differences in the composition of the immune system, insufficient genome annotation, and the lack of related databases, which brings difficulties for the application of single-cell omics technology in chickens. Previously, we explored the in vivo heterogeneity of duck blood immune cells in homeostasis and following flaviviral infection using scRNA-seq [15]. This work constructed the first transcriptomic landscape of avian immune cells and highlighted the importance of granulocytes and monocytes in the duck’s response to flaviviral infection. In the present study, we monitored the dynamic transition of immune cells in peripheral blood within two weeks post-vaccination with the widely used live attenuated IBV vaccine strain H120 using scRNA-seq, which illustrated the initiation of immune responses in chickens against IBV infection.

## 2. Materials and Methods

### 2.1. Ethics Statement

The design of animal experiments and the use of peripheral blood cells from chickens were approved by the Animal Ethics Committee of Harbin Veterinary Research Institute (HVRI) of the Chinese Academy of Agricultural Sciences (CAAS).

### 2.2. Animal Experiments and Virus

Healthy 35-day-old male specific-pathogen free (SPF) chickens were obtained from and kept at the National Poultry Laboratory Animal Resource Center. The IBV strain H120 (G1–1 lineage), which has been widely used as a commercial live attenuated vaccine for prime vaccination worldwide, was stored at the HVRI. This strain is propagated in SPF chicken embryos. For live vaccine immunization, chickens received 100 microliters (μL) of virus specimens at 10^4^ EID_50_ per milliliter (mL) of live H120 through the nasal route. Peripheral blood was taken from these chickens at 0, 4, 7, 14 days post-vaccination (dpv) and transferred to the laboratory for further treatment within one hour.

### 2.3. Isolation of Peripheral Blood Mononuclear Cells

Ten milliliters of peripheral blood taken from each chicken was pretreated with erythrocyte lysis buffer (SolelyBio, Shanghai, China) and separated by density gradient centrifugation with a chicken lymphocyte isolation kit (Ficoll; Haoyang Biological Technology, Tianjin, China). Peripheral blood mononuclear cells (PBMCs) located in the middle layer were washed with RPMI 1640 culture medium (Gibco, Carlsbad, CA, USA) and the cell concentrations adjusted to 1 × 10^6^ cells/mL for subsequent analysis.

### 2.4. Serology Detections

Serum samples were collected from all chickens at indicated timepoints, and the clots were separated to obtain the serum. The levels of antibody specifically recognizing viral N protein, the most plentiful viral protein during viral replication, were determined using a commercial IBV enzyme-linked immunosorbent assay kit (NECVB, Harbin, China). The sample-to-positive (S/P) ratio was calculated. A serum sample with S/P ratios less than or equal to 0.20 was considered negative; a S/P ratio greater than 0.20 was considered positive. The levels of IBV-neutralizing antibody were determined by virus neutralization tests through observing the reduction in viral infectivity based on the absence of cytopathic effects on embryonated eggs.

### 2.5. RNA Sequencing

The scRNA-seq procedure was performed as previously described [15]. One million PBMCs taken from each sample were mixed together. Then, 10,000 pooled cells were processed for constructing the libraries following the user guide of Chromium™ Single Cell 3′ Reagent Kits v2 using the Chromium™ Single Cell Controller (10× Genomics, Pleasanton, CA, USA). The Illumina HiSeq 2500 system was used for subsequent sequencing. The cell viabilities of these samples were greater than 90%.

### 2.6. Transcriptome Data Analysis

For the analysis of single-cell transcriptome data, the complementary DNA reads were aligned to chicken reference (assembly: GRCg6a) with Cell Ranger pipelines (version 3.0.1, https://github.com/10XGenomics/cellranger, accessed on 11 March 2022). More than 90% of the reads were mapped to the assembly GRCg6a for all data. Default parameters were used to generate the expression matrix of a filtered unique molecular index (UMI). The “cellranger aggr” function was conducted for the normalization of expression matrix in the Cell Ranger. The abnormal cells were filtered out according to their gene expression distribution based on Cell Ranger pipelines with the following criteria: (i) detected gene number < 200; (ii) detected gene number > 2500; and (iii) less than 10% of detected genes were mitochondrial genes. A “detected gene” is defined as any gene expressed in ≥3 individual cells at a level of UMI ≥ 1. Raw data of scRNA-seq were uploaded to the National Center for Biotechnology Information database (GSE220071).

### 2.7. Clustering and Cell-Cell Communication Analysis

The Seurat R toolkit (version 3.1.5, https://github.com/satijalab/seurat, accessed on 20 January 2023) was used for unsupervised cell clustering following the standard pipeline [16]. The uniform manifold approximation and projection (UMAP) or t-distributed stochastic neighborhood embedding (t-SNE) was performed to visualize the cell composition landscape of chicken peripheral blood in 2D space. Cell types were annotated according to the expression of well-known marker genes manually [17,18,19,20,21,22,23]. The information on orthologous genes was downloaded from Ensembl BioMart [24]. The CellChat R toolkit (version 1.4.0) [25] was used for cell–cell communication analysis using chicken–human orthologous genes.

### 2.8. Statistical Analysis

The SPSS software package (Version 13.0, SPSS Inc., Chicago, IL, USA) was used for statistical analyses. Data obtained from several experiments are presented as mean ± standard deviation. The significance of differences between two groups was determined with the two-tailed Student’s *t*-test, assuming a normal distribution and equal variances. For all analyses, a probability (*p*) value of < 0.05 was considered statistically significant.

## 3. Results

### 3.1. Experimental Model and Dynamic PBMC Landscapes Responding to Prime Vaccination with Live Attenuated IBV Vaccine

**Figure 1:**
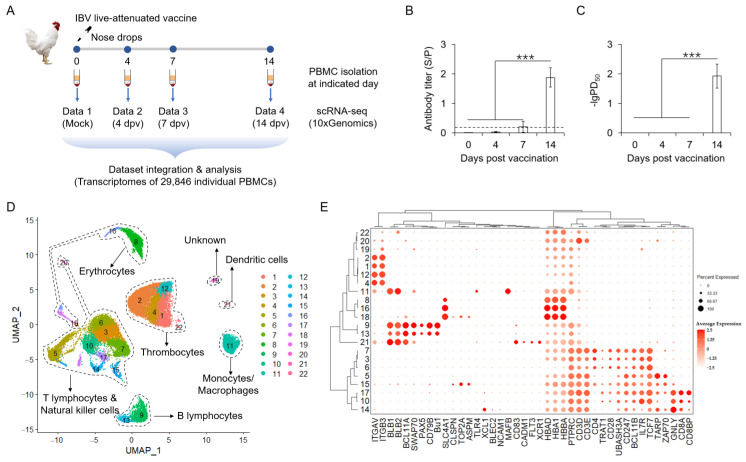
Constructing the dynamic transcriptomic landscape of chicken PBMCs upon IBV vaccination. (**A**) Illustration of the experimental workflow. (**B**) Detection of antibodies targeting viral N protein. *n* = 6. (**C**) Detection of serum-neutralizing antibody. *n* = 6. (**D**) Uniform manifold approximation and projection (UMAP) clustering of chicken PBMCs. Cells of each cluster are labelled by different colors. (**E**) Dot plots of the expression of chicken immune cell markers. scRNA-seq: Single-cell RNA sequencing, dpv: days post-vaccination, ***: *p* < 0.001.

Thirty-five-day-old specific-pathogen-free (SPF) male chickens were inoculated with the live attenuated vaccine strain H120 (six half-sibs per group). Blood samples were collected at the onset of vaccination and at 4, 7, and 14 days post-vaccination (dpv) as indicated in Figure 1A. Following vaccination, an increase in antibodies specifically recognizing the viral N protein, the most abundant viral protein during replication, was observed from 7 dpv (positive in two of six chickens) and significantly induced in all chickens by 14 dpv (Figure 1B). Consistently, serum-neutralizing antibodies were only observed at 14 dpv in immunized chickens (Figure 1C), while neither antibody specifically recognizing viral N protein nor IBV-neutralizing antibody was observed at 0 dpv, suggesting the absence of antiviral antibodies and IBV infection in these SPF chickens before vaccination (Figure 1B,C). These findings suggest successful vaccination in our in vivo model.

The four scRNA-seq data from the PBMCs of chickens at the onset of inoculation and 4, 7, and 14 dpv were generated as indicated in Figure 1A. High-quality transcriptomes of 29,846 PBMCs with medians of 1154 UMI reads and 601 genes per cell were obtained based on the following quality control criteria: (i) detected gene number between 200 and 2500; (ii) a proportion of mitochondrial genes in detected genes < 10%; and (iii) all “detected genes” expressed in ≥ 3 individual cells at a level of UMI ≥ 1. Globally, unsupervised cell clustering using Seurat identified 22 clusters, as shown in the 2D space by UMAP (Figure 1D). Cells were annotated according to the transcription level of known marker genes manually (Figure 1E). Four classic erythrocyte markers, HBA1, HBAD, HBBA, and SLC4A1, were expressed at high levels in clusters (C) 8, 16, and 18, suggesting that these three clusters consisted of residual erythrocytes which were removed from subsequent analysis. The thrombocyte markers ITGAV and ITGB3 were expressed at high levels in C1, 2, 4, and 12, which assigned these four clusters as thrombocytes. The genes encoding class II histocompatibility antigens, BLB1 and BLB2, were highly expressed in four clusters, C9, 11, 13, and 21. Among these clusters, the B cell markers BCL11A, SWAP70, PAX5, CD79B, and Bu-1 were expressed at high levels in C9 and C13, which assigned these cells as B lymphocytes. Monocyte markers TLR4 and MAFB were enriched in C11, and dendritic cell markers CADM1, FLT3, and XCR1 were enriched in C21, assigning these two clusters as monocytes and dendritic cells, respectively. C10 and C17 were assigned as CD8+ T lymphocytes, giving the expression of CD8A and CD8BP. C3, 6, and 17 were assigned as CD4+ T lymphocytes, giving the expression of CD4 and CD28. Cluster C5 was considered as γδT lymphocytes, given the high expression of TCR gamma TARP and ZAP70. Giving the high expression of CD3D in C20, this cluster was assigned as T lymphocytes too. Considering the high levels of GNLY, NCAM1 (CD56), BLEC2, and XCL1 in C14 and the high levels of CLSPN, TOP2A, and ASPM in C15, these two clusters highly expressing PTPRC, CD3D, and CD3E were either T lymphocytes or natural killer (NK) cells.

### 3.2. Overview of Cell–Cell Communication Network of PBMCs upon Vaccination

**Figure 2:**
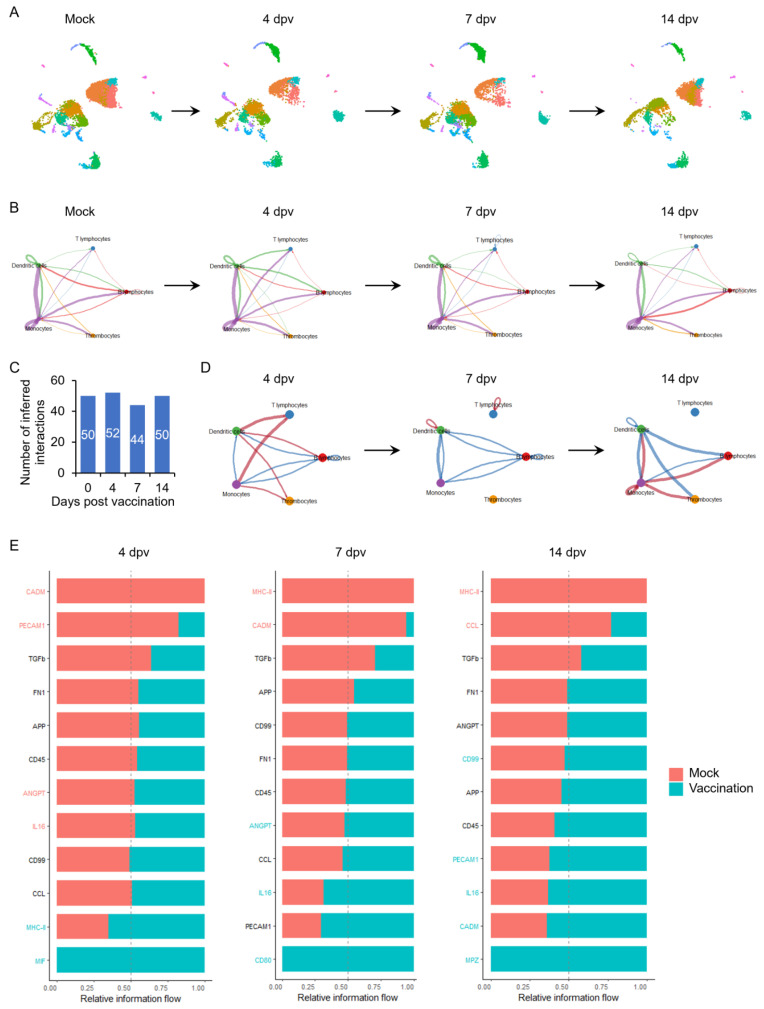
Dynamic PBMC landscapes and overview of cell–cell communication network upon vaccination. (**A**) Transition of chicken PBMC composition upon vaccination. (**B**) The dynamic cell–cell communication network upon vaccination was inferred using CellChat (version 1.4.0). Circle plots presenting the network centrality analysis of the signaling pathways transduced among PBMCs within two weeks post-vaccination. Different colors represent different cell types, and edge width is proportional to the communication probability. Arrows and edge color indicate direction (source: target). (**C**) The numbers of inferred interactions at indicated days post-vaccination were summarized. (**D**) Comparison of the cell–cell communication of PBMCs post-vaccination with those at the onset of vaccination is presented with circle plots. Different colors represent different cell types, and edge width is proportional to the communication probability. Arrows indicate direction (source: target). Edge color: red indicates promotion, and blue indicates reduction. (**E**) Signaling in red font indicates more enrichment in the mock group, while signaling in blue font indicates more enrichment in the vaccination group. Signaling equally enriched in both groups is colored black. dpv: days post-vaccination.

Vaccination significantly reshaped the composition of PBMCs as early as 4 dpv (Figure 2A). The cell–cell communication network of host PBMCs was inferred and analyzed using CellChat (version 1.4.0) with label-based mode (Figure 2B). The total number of inferred interactions among different types of PBMCs increased from 50 ligand–receptor pairs to 52 at 4 dpv, decreased to 44 at 7 dpv, and finally returned to 50 at 14 dpv (Figure 2C). Monocytes were identified as the dominant communication “hub” secreting and receiving more signals via ligand–receptor pairs than other cells throughout the observation period (Figure 2B). To elucidate the dynamic transition of the cell–cell communication network of host PBMCs upon vaccination, the difference in the number of interactions among PBMCs compared to those at the onset of vaccination was calculated and presented in Figure 2D, with red lines indicating an increase in interaction number and blue lines indicating a decrease. At 4 dpv, T lymphocytes received more abundant signals from the main antigen-presenting cells (APCs), including monocytes and dendritic cells, compared to the cellular communications between T lymphocytes and these APCs before vaccination (Figure 2D, left panel). This finding aligns with the enhanced MHC class II signal among PBMCs at 4 dpv (Figure 2E, left panel), suggesting the activation of T lymphocytes by APCs at this early stage post-vaccination. The promoted interactions between T lymphocytes and APCs were quickly replaced by auto-interactions within T lymphocytes at 7 dpv (Figure 2D, middle panel), along with a reduction in the MHC class II signal (Figure 2E, middle panel), indicating the subsequent activation and differentiation of specific subpopulations within T lymphocytes upon initial activation by APCs. The signals received by T lymphocytes returned to baseline levels before vaccination at 14 dpv (Figure 2D, right panel), at which point viral-specific antibodies were significantly induced (Figure 1B,C). Meanwhile, all signals sent by monocytes were reduced, while all signals received by monocytes were enhanced (Figure 2D, right panel). Along with the MHC class II signal, the MIF signal was also only promoted at 4 dpv (Figure 2E). IL-16 and CADM signals were repressed at 4 dpv but consistently promoted after 7 dpv. ANGPT and PECAM1 signals were reduced at 4 dpv but enhanced at 7 and 14 dpv, respectively.

**Figure 3:**
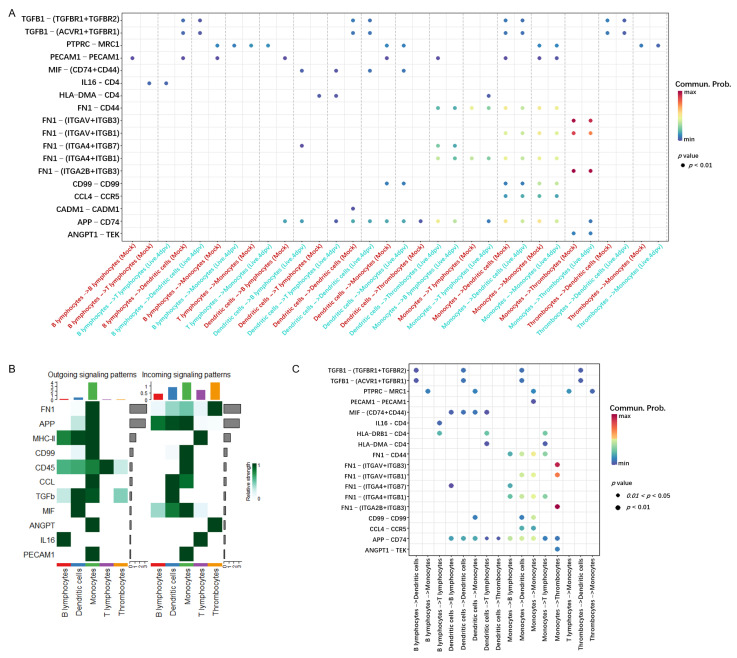
Identification of major signaling changed at 4 days post-vaccination. (**A**) Significant ligand–receptor pairs between PBMCs before and after vaccination. The communication probabilities were reflected with the color of dot color, and the computed *p*-values were reflected with the size of dot. The communication probability of zero was presented as empty space. One-sided permutation test was used to compute *p*-values. (**B**,**C**) Comparison of these significant ligand–receptor pairs between each cell type, presented in a heatmap (**B**) and dot plot (**C**), respectively.

Given the importance of cell–cell communication among PBMCs at 4 dpv in initiating antiviral adaptive immune responses (Figure 2D, left panel), we compared the intercellular communication patterns of 18 significant ligand–receptor pairs between specific types of PBMCs from the onset of vaccination to 4 dpv, as visualized in Figure 3A. The comparison results are shown in Figure 3B,C. These ligand–receptor pairs belong to 11 signaling pathways, with FN1, APP, and MHC class II identified as the top three strongest signals (Figure 3B). Monocytes were identified as the main source of these three signals. Thrombocytes were the primary receivers of the FN1 signal, while APP signals could be received by all major APCs. There were five significant receptors in the FN1 signal, but only the communications between FN1 and three of them, namely ‘ITGAV + ITGB3’, ‘ITGAV + ITGB1’, and ‘ITGAB + ITGB3’, were enhanced between monocytes and thrombocytes (Figure 3C). Consistent with Figure 3B, the ‘APP-TEK’ ligand–receptor pair was mostly activated between monocytes and all APCs, including a paracrine signal loop within monocytes. Besides monocytes, the MHC class II signal was also sent by other APCs, with T lymphocytes being the only receivers.

Together, the aforementioned joint analysis facilitates the discovery of significant signaling network changes that may drive the initiation of antiviral adaptive immune responses following inoculation with the IBV live attenuated vaccine.

### 3.3. Global Intercellular Communication of Major APCs During the Initiation of Antiviral Adaptive Immune Response

**Figure 4:**
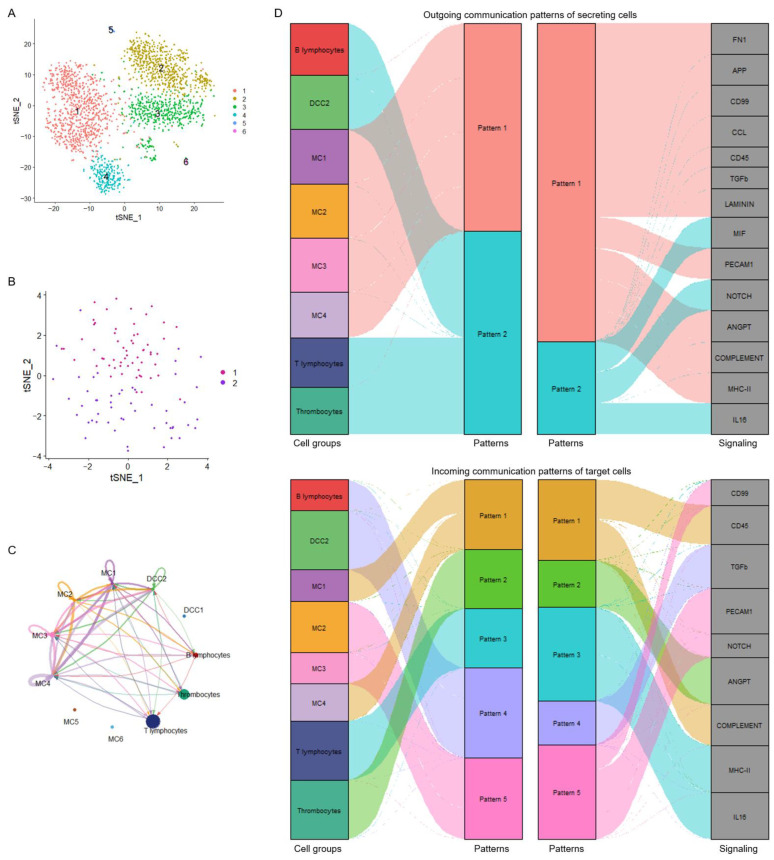
Global intercellular communication of major APCs during the initiation of antiviral adaptive immune response. (**A**) t-distributed stochastic neighbor embedding (t-SNE) clustering of chicken monocytes. Each subcluster of monocytes is labelled by different colors. (**B**) t-SNE clustering of chicken dendritic cells. Each subcluster of dendritic cells is labelled by different colors. (**C**) Circle plots presenting the overall cell–cell communication network among each subcluster of APCs and other PBMCs. Different colors represent different cell types, and edge width is proportional to the communication probability. Arrows and edge color indicate direction (source: target). (**D**) The deduced communication patterns of secreting cells and the reception patterns of target cells illustrate the relationship between the inferred latent patterns and cell groups, along with the signaling pathways. The flow’s thickness represents the extent of contribution from each cell group or signaling pathway to the latent pattern.

**Figure 5:**
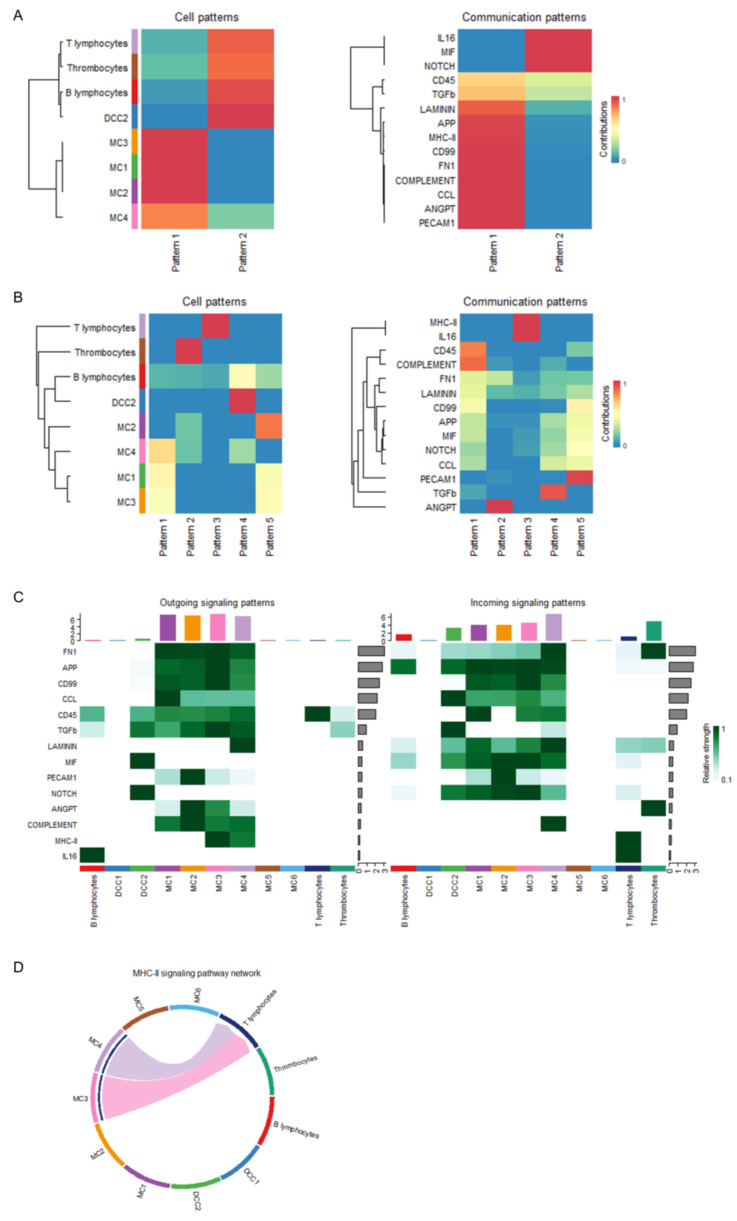
Dynamic transition of the global intercellular communication of major APCs at 4 days post-vaccination. (**A**,**B**) Heatmaps presenting the coordination of cells and signaling based on outgoing communication patterns of secreting cells (**A**) and incoming communication patterns of target cells (**B**). (**C**) Heatmap presenting the comparison of significant signaling between each cell type. (**D**) Circle plots presenting the inferred MHC class II signaling network centrality analysis of the signaling pathways transduced among PBMCs at 4 days post-vaccination. Different colors represent different cell types. Arrows and edge color indicate direction (source: target).

Given that major antigen-presenting cells (APCs), such as monocytes and dendritic cells, were identified as the dominant communication “hubs” during the initiation of antiviral adaptive immune responses, the intercellular communication networks of monocytes and dendritic cells at 4 dpv were analyzed in detail. Monocytes were reclustered and grouped into six subclusters (MC1–6; Figure 4A). A few dendritic cells were obtained and defined as two subclusters, DCC1 and DCC2 (Figure 4B). The overview of the cell–cell communication network among these clusters and other PBMCs was inferred and analyzed (Figure 4C). Among these APC subclusters, MC5, MC6, and DCC1 were not involved in the PBMC communication network at 4 dpv. To explore how multiple APC subclusters and signaling pathways coordinate the initiation of antiviral adaptive immune responses, the global communication patterns during this process were analyzed using a pattern recognition method based on non-negative matrix factorization. Figure 4D presents the communication patterns connecting cell groups with signaling pathways. The cells connected with outgoing signaling or incoming signaling were recognized as senders or receivers, respectively. Two patterns for outgoing signaling (upper panel) and five patterns for incoming signaling (lower panel) were revealed. The outgoing signaling of all monocyte subclusters was characterized by pattern 1, which represents multiple pathways, including but not limited to MHC class II, complement, CD45, TGF-β, and CCL, demonstrating the simultaneous activation of multiple signaling pathways by monocytes during the initiation of antiviral adaptive immune responses. The outgoing signaling of other PBMCs, including DCC2, T lymphocytes, B lymphocytes, and thrombocytes, was characterized by pattern 2, representing pathways such as MIF, NOTCH, and IL-16, indicating the coordination of these cells at this stage. On the other hand, the communication patterns of target cells (Figure 4D, lower panel) showed more homogeneous communication patterns. The incoming monocyte signaling was dominated by patterns 1 and 5, which include signaling pathways such as complement, CD45, C99, NOTCH, and PECAM1. Except for NOTCH signaling, all of these signals were sent out by monocytes themselves, suggesting abundant internal communication among monocyte subclusters and autocrine signal transduction. The signaling pathways of pattern 3, including MHC class II and IL-16, were only received by T lymphocytes, suggesting a unique transduction of MHC class II signaling from monocytes to T lymphocytes. TGF-β signaling was the only signal transduced exclusively among APCs, sent out by monocytes and received by B lymphocytes and DCC2, indicating potential control of B lymphocytes and dendritic cells by monocytes during the initiation of adaptive immunity. Thrombocytes only received ANGPT signaling sent out by monocytes. Similarity measures and manifold learning revealed MC1–4 as the common sources of signaling in pattern 1 of outgoing signaling (Figure 5A, left panel), with high similarity among signaling in pattern 1 (Figure 5A, right panel). Other major APCs, namely dendritic cells and B lymphocytes, were closely grouped together. For incoming cells and pathways, MC1, 3, and 4 were identified as the main sources of signaling in patterns 1 and 5, while B lymphocytes were shown as a broad but generally weak source of all incoming signaling (Figure 5B, left panel). The signaling in patterns 1 and 5 exhibited highly coordinated patterns, indicating complex intercellular communication networks among different subclusters of monocytes (Figure 5B, right panel). Further quantitative analysis of these intercellular communications confirmed monocytes as the predominant source of outgoing signaling during the initiation of antiviral adaptive immunity, as all of the signaling received by other PBMCs was sent out by monocytes (Figure 5C). Among the four subclusters of monocytes involved in triggering adaptive immune responses, the MHC class II pathway was dominantly secreted by MC3 and MC4 (Figure 5D).

### 3.4. Global Intercellular Communication of T Lymphocytes During the Initiation of Antiviral Adaptive Immune Response

**Figure 6:**
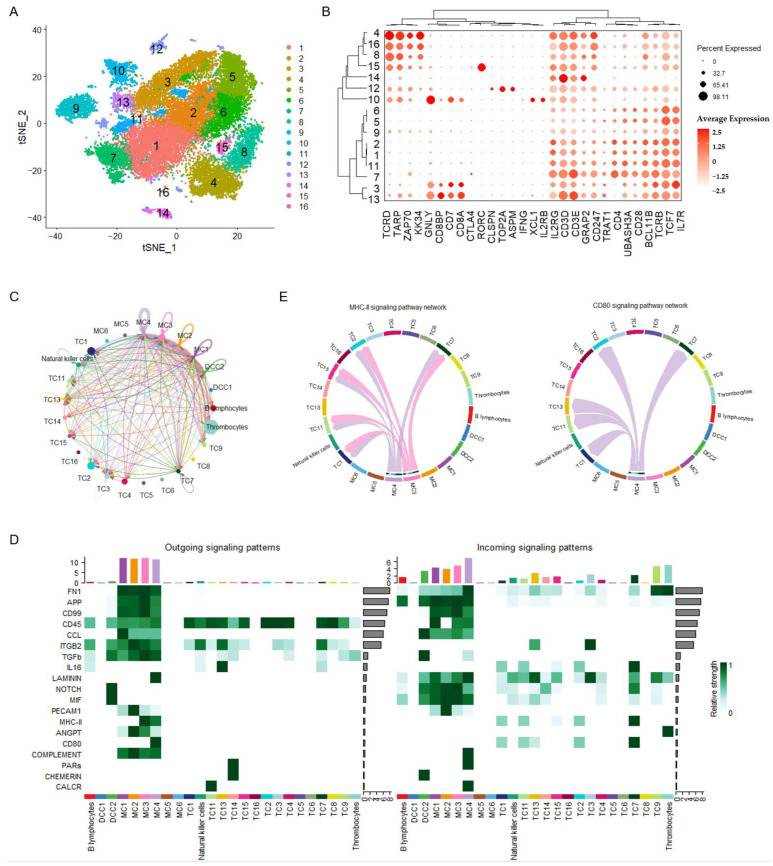
Global intercellular communication of T lymphocytes during the initiation of antiviral adaptive immune response. (**A**) t-distributed stochastic neighbor embedding (t-SNE) clustering of chicken T lymphocytes. Each subcluster of T lymphocytes is labeled with different colors. (**B**) Dot plots showing the expression of chicken T lymphocyte and natural killer cell markers. (**C**) Circle plots presenting the overall cell–cell communication network among each subcluster of APCs and T lymphocytes. Different colors represent different cell types, and edge width is proportional to the communication probability. Arrows and edge color indicate direction (source: target). (**D**) Heatmap presenting the comparison of significant signaling between each cell type. (**E**) Circle plots presenting the inferred MHC class II signaling network and CD80 signaling network centrality analysis of the signaling pathways transduced among PBMCs at 4 days post-vaccination. Different colors represent different cell types. Arrows and edge color indicate direction (source: target).

To explore the biological significance of APC preference for T lymphocyte activation upon vaccination, T lymphocytes were reclustered, identifying 16 clusters, indicating substantial cellular heterogeneity (TC1–16; Figure 6A). Using 29 known chicken T lymphocyte and natural killer cell markers detectable in our data, combined with developmental clustering using transcriptional profiles, these 16 clusters were defined as CD4+ T lymphocytes (TC1, 2, 5, 6, 7, and 11), CD8+ T lymphocytes (TC3 and 13), γδT lymphocytes (TC4, 8, 15, and 16), other T lymphocytes (TC9 and 14), and natural killer cells (TC10 and 12) (Figure 6A,B). The overview of the cell–cell communication network among these subclusters of monocytes, T lymphocytes, and other PBMCs was inferred and analyzed (Figure 6C). Among the 16 T lymphocyte subclusters, two subclusters of CD4+ T lymphocytes, namely TC5 and TC6, and a subcluster of γδT lymphocytes, TC16, were not involved in the initiation of adaptive immune responses, as there were no signal interactions sent or received by these cells. The cell–cell communication network of T lymphocyte activation upon vaccination was elucidated by analyzing the signaling transduction between APCs and each T lymphocyte subcluster (Figure 6D). Most of the signaling sent by T lymphocytes, including CD45, TGF-β, PARs, CHEMERIN, and CALCR signaling, was transduced to monocytes and dendritic cells. ITGB2 was received by CD8+ T lymphocytes (TC3 and 13), and IL-16 signaling was received by CD4+ T lymphocytes (TC1, 2, 7, and 11). T lymphocyte activation upon vaccination was elucidated by identifying signaling from APCs to T cells via the MHC class II and CD80 (B7) signaling pathways at 4 dpv (Figure 6D). The MHC class II signaling was sent by both MC3 and MC4 and received by CD4+ T lymphocytes (TC1, 2, 7, and 11) and γδT lymphocytes (TC15) (Figure 6E, left panel). CD80 signaling was also received by CD4+ T lymphocytes (TC1, 2, 7, and 11) but only sent by MC4 (Figure 6E, right panel). Considering the pivotal role of CD80 in CD4+ T lymphocyte activation as a coactivator, MC4 may play a central role in the initiation of antiviral adaptive immune responses upon IBV vaccination.

## 4. Discussion

Understanding the initiation of adaptive immune responses by prime vaccination is essential for the rational design of novel, biosafe, and effective vaccines. Despite significant efforts to elucidate host immune responses upon coronavirus infection and vaccination, our comprehensive understanding of coronavirus prime vaccination remains limited, especially for avian coronaviruses. IBV, the first discovered coronavirus, poses a major threat to the global poultry industry and serves as a prototype for coronaviruses. A single immunization with the live attenuated IBV vaccine is sufficient to elicit satisfactory antiviral adaptive immune responses in the host (Figure 1B,C). Utilizing this model, we constructed in vivo dynamic immune landscapes of prime vaccination with the live attenuated IBV vaccine at single-cell resolution. Vaccination dramatically altered the host immune landscape within the first week post-immunization, reshaping PBMCs into an immune activation composition by two weeks (Figure 2). This rapid and dramatic change is likely the result of the infectious bronchitis virus evading the host’s innate immune system through multiple mechanisms via its nonstructural proteins, allowing the virus to replicate quickly without any innate defense from the host [26]. This is beneficial for the rapid initiation of antiviral adaptive immunity and may reflect the superiority of live attenuated vaccines. The immune responses elicited by the live attenuated IBV vaccine correlate with the enhanced interaction between monocytes and T lymphocytes and subsequent T lymphocyte activation, which appears essential for effective prime vaccination in chickens (Figure 3, Figure 4 and Figure 5). Our findings underscore the important cell–cell communication network and key cell populations during the initiation of successful immune responses in chickens against IBV infection, which may also be valuable for the rational design of safe and effective vaccines against IBV.

Live attenuated vaccines have been extensively administered for the prevention and control of infectious diseases worldwide due to their superior protection [8,9]. However, growing evidence has raised concerns about the biosafety risks associated with using live viruses as vaccines for a broad spectrum of viruses, such as polioviruses, coronaviruses, herpesviruses, and influenza viruses, which may lead to the constant emergence of new variants [8,9,27]. Our analysis revealed the transduction of MHC class II signaling from monocytes to CD4+ T lymphocytes at the early initiation stage of antiviral adaptive immune responses upon live IBV vaccination (Figure 4, Figure 5 and Figure 6). This capability to elicit antiviral immune responses suggests that the preference for monocytes as APCs may be essential for effective prime vaccination in chickens and indicates the possibility of enhancing the effectiveness of other types of vaccines, such as inactivated IBV vaccines, through APC switching. Many efforts have been made to address why live virus vaccines are more effective than inactivated vaccines in most cases. Most of these studies have focused on differences in lymphocyte composition after vaccination. Contrary to our expectations, our analysis observed monocytes as the preferred APCs at the onset of successful adaptive immune response initiation. Additional analysis of prime vaccination for more pathogens in various species is necessary to determine the universality of our findings.

In addition to MHC class II signaling sent by monocytes, MIF signaling was also significantly promoted by vaccination at the initiation stage of adaptive immune response activation (Figure 2E). Further analysis of the ‘MIF − (CD74 + CD44)’ ligand–receptor pair revealed that this signaling was sent by dendritic cells and mainly received by other APCs, especially monocytes (Figure 3). MIF − (CD74 + CD44) has been previously reported to function as a recruiter of immunosuppressive cells, thus promoting immunosuppression [28,29,30]. Considering the important role of peripheral blood monocytic cells during IBV dissemination and kidney infection in chickens [31,32], the enhanced MIF signaling among APCs may be a strategy for the live IBV vaccine strain H120 to evade immune surveillance during its efficient initiation of antiviral adaptive immune responses.

To investigate immune dynamics following IBV vaccination, peripheral blood immune cells, which conveniently reflect systemic changes in immune responses, were extensively monitored using scRNA-seq analysis in this study. This approach enabled time series observations under a paired experimental design. However, the results from the analysis of systemic immune dynamics may not accurately represent the primary mechanisms and mediators of effective immune responses against IBV in the mucosal compartment of vaccinated chickens, where antiviral adaptive immune responses are likely initially triggered by the live attenuated vaccine. Given the importance of both mucosal and systemic compartments in effective immune responses against IBV, the parameters identified in the current analysis need to be assessed within the tracheal mucosal immune compartment in future research.

In an industrial-scale system, all chicks are typically prime vaccinated just after hatching or during the first week of age with live attenuated IBV vaccines, such as the H120 vaccine. Given that all chicks have maternal antibodies, the phenomenon of maternal antibody interference must be taken into account. The presence of maternal antibodies may inhibit the generation of new antibodies by B lymphocytes in chicks through epitope masking, direct binding of maternal antibodies to the H120 vaccine, which prevents it from being recognized by the chick’s immune system, and/or B lymphocyte inhibition through the cross-linking of BCR with the Fcγ-receptor via the maternal antibody-H120 complex [33,34,35,36]. In addition to these maternal antibody feedback mechanisms, the removal of vaccine antigen by macrophages and neutralization of the vaccine virus by maternal antibodies have been hypothesized to play a role in maternal antibody-mediated inhibition of vaccination, but with little experimental evidence and controversial conclusions [37,38]. Despite the inhibition of the humoral immune response, the cell-mediated immune response mediated by T lymphocytes is usually unaffected by maternal antibodies [37,38]. The partial immune response initiated by prime vaccination can still provide some level of protection against IBV, but it may not be as robust as in chicks without maternal antibodies. Thus, in the context of the PBMC landscape, maternal antibodies may affect the activation and proliferation of B lymphocytes, but the landscape of the cell-mediated immune response may not be affected. However, the antiviral adaptive immune responses initiated by vaccination are not only determined by the shift of cellular composition of immune cells but also rely on the reformed immune repertoires. It is possible that maternal antibodies might reshape immune repertoires differently upon prime vaccination through epitope masking. Further single-cell transcriptome analysis in combination with immune repertoire analysis in chicks with or without maternal antibodies upon prime vaccination may address this issue properly.

## 5. Conclusions

A comprehensive understanding of IBV vaccination is essential for the rational design of novel, effective, and safe vaccines. Our present study provides single-cell in vivo dynamic immune landscapes of vaccination against one of the major respiratory infectious diseases in chickens and highlights the critical cell–cell communication networks and key cell populations involved in the effectiveness of vaccination. This may contribute to further investigations into the mechanisms underlying successful prime vaccination in birds and could serve as a standard dataset for comparative analysis across species.

## Data Availability

Raw RNA sequencing data were uploaded to the National Center for Biotechnology Information database under the accession number GSE220071. All additional data included in this study are available from the corresponding authors upon request. No special code was reported in this paper.

## References

[1] Rafique S., Jabeen Z., Pervaiz T., Rashid F., Luo S., Xie L., Xie Z. (2024). Avian infectious bronchitis virus (AIBV) review by continent. Front. Cell Infect. Microbiol..

[2] Schalk A.F., Hawn M.C. (1931). An apparently new respiratory disease in baby chicks. J. Am. Vet. Med. Assoc..

[3] Beaudette F., Hudson C. (1937). Cultivation of the virus of infectious bronchitis. J. Am. Vet. Med. Assoc..

[4] Cavanagh D. (2003). Severe acute respiratory syndrome vaccine development: Experiences of vaccination against avian infectious bronchitis coronavirus. Avian Pathol..

[5] Nefedova E., Koptev V., Bobikova A.S., Cherepushkina V., Mironova T., Afonyushkin V., Shkil N., Donchenko N., Kozlova Y., Sigareva N. (2021). The Infectious Bronchitis Coronavirus Pneumonia Model Presenting a Novel Insight for the SARS-CoV-2 Dissemination Route. Vet. Sci..

[6] Franzo G., Tucciarone C.M., Blanco A., Nofrarías M., Biarnés M., Cortey M., Majó N., Catelli E., Cecchinato M. (2016). Effect of different vaccination strategies on IBV QX population dynamics and clinical outbreaks. Vaccine.

[7] Zhang X.R., Guo M.J., Zhao J., Wu Y.T. (2021). Avian Infectious Bronchitis in China: Epidemiology, Vaccination, and Control. Avian Dis..

[8] Zepp F. (2016). Principles of Vaccination. Methods Mol. Biol..

[9] Vetter V., Denizer G., Friedland L.R., Krishnan J., Shapiro M. (2018). Understanding modern-day vaccines: What you need to know. Ann. Med..

[10] Guzman E., Montoya M. (2018). Contributions of Farm Animals to Immunology. Front. Vet. Sci..

[11] Silva A.P.D., Gallardo R.A. (2020). The Chicken MHC: Insights into Genetic Resistance, Immunity, and Inflammation Following Infectious Bronchitis Virus Infections. Vaccines..

[12] Yang W., Liu X., Wang X. (2023). The immune system of chicken and its response to H9N2 avian influenza virus. Vet. Q..

[13] Papalexi E., Satija R. (2018). Single-cell RNA sequencing to explore immune cell heterogeneity. Nat. Rev. Immunol..

[14] Chen H., Ye F., Guo G. (2019). Revolutionizing immunology with single-cell RNA sequencing. Cell. Mol. Immunol..

[15] Liang Y., Ma Y., Zhang Y., Chen Z., Wang Z., Li X., Cui L., Xu L., Liu S., Li H. (2021). Single-cell analysis of the in vivo dynamics of host circulating immune cells highlights the importance of myeloid cells in avian flaviviral infection. J. Immunol..

[16] Stuart T., Butler A., Hoffman P., Hafemeister C., Papalexi E., Mauck W.M., Hao Y., Stoeckius M., Smibert P., Satija R. (2019). Comprehensive integration of single-cell data. Cell.

[17] Houssaint E., Lassila O., Vainio O. (1989). Bu-1 antigen expression as a marker for B cell precursors in chicken embryos. Eur. J. Immunol..

[18] Nera K.P., Kohonen P., Narvi E., Peippo A., Mustonen L., Terho P., Koskela K., Buerstedde J.M., Lassila O. (2006). Loss of Pax5 promotes plasma cell differentiation. Immunity.

[19] Viertlboeck B.C., Göbel T.W. (2007). Chicken thrombocytes express the CD51/CD61 integrin. Vet. Immunol. Immunopathol..

[20] Vu Manh T.P., Marty H., Sibille P., Le Vern Y., Kaspers B., Dalod M., Schwartz-Cornil I., Quéré P. (2014). Existence of conventional dendritic cells in Gallus gallus revealed by comparative gene expression profiling. J. Immunol..

[21] You Z., Zhang Q., Liu C., Song J., Yang N., Lian L. (2019). Integrated analysis of lncRNA and mRNA repertoires in Marek’s disease infected spleens identifies genes relevant to resistance. BMC Genom..

[22] Hao X., Li S., Chen L., Dong M., Wang J., Hu J., Gu M., Wang X., Hu S., Peng D. (2020). Establishing a Multicolor Flow Cytometry to Characterize Cellular Immune Response in Chickens Following H7N9 Avian Influenza Virus Infection. Viruses.

[23] Wu Z., Hu T., Chintoan-Uta C., Macdonald J., Stevens M.P., Sang H., Hume D.A., Kaiser P., Balic A. (2022). Development of novel reagents to chicken FLT3, XCR1 and CSF2R for the identification and characterization of avian conventional dendritic cells. Immunology.

[24] Martin F.J., Amode M.R., Aneja A., Austine-Orimoloye O., Azov A.G., Barnes I., Becker A., Bennett R., Berry A., Bhai J. (2023). Ensembl 2023. Nucleic Acids Res..

[25] Jin S., Guerrero-Juarez C.F., Zhang L., Chang I., Ramos R., Kuan C.H., Myung P., Plikus M.V., Nie Q. (2021). Inference and analysis of cell-cell communication using CellChat. Nat. Commun..

[26] Peng S., Wang Y., Zhang Y., Song X., Zou Y., Li L., Zhao X., Yin Z. (2022). Current Knowledge on Infectious Bronchitis Virus Non-structural Proteins: The Bearer for Achieving Immune Evasion Function. Front. Vet. Sci..

[27] Thomas S., Abraham A., Rodríguez-Mallon A., Unajak S., Bannantine J.P. (2022). Challenges in Veterinary Vaccine Development. Methods Mol. Biol..

[28] Schwartz V., Lue H., Kraemer S., Korbiel J., Krohn R., Ohl K., Bucala R., Weber C., Bernhagen J. (2009). A functional heteromeric MIF receptor formed by CD74 and CXCR4. FEBS Lett..

[29] Borghese F., Clanchy F.I. (2011). CD74: An emerging opportunity as a therapeutic target in cancer and autoimmune disease. Expert. Opin. Ther. Targets.

[30] Gore Y., Starlets D., Maharshak N., Becker-Herman S., Kaneyuki U., Leng L., Bucala R., Shachar I. (2008). Macrophage migration inhibitory factor induces B cell survival by activation of a CD74-CD44 receptor complex. J. Biol. Chem..

[31] Reddy V.R., Trus I., Desmarets L.M., Li Y., Theuns S., Nauwynck H.J. (2016). Productive replication of nephropathogenic infectious bronchitis virus in peripheral blood monocytic cells, a strategy for viral dissemination and kidney infection in chickens. Vet. Res..

[32] Amarasinghe A., Abdul-Cader M.S., Nazir S., De Silva Senapathi U., van der Meer F., Cork S.C., Gomis S., Abdul-Careem M.F. (2017). Infectious bronchitis corona virus establishes productive infection in avian macrophages interfering with selected antimicrobial functions. PLoS ONE.

[33] Getahun A., Heyman B. (2009). Studies on the mechanism by which antigen-specific IgG suppresses primary antibody responses: Evidence for epitope masking and decreased localization of antigen in the spleen. Scand. J. Immunol..

[34] Sinclair N.R., Lees R.K., Elliott E.V. (1968). Role of the Fc fragment in the regulation of the primary immune response. Nature.

[35] Karlsson M.C., Wernersson S., Diaz de Ståhl T., Gustavsson S., Heyman B. (1999). Efficient IgG-mediated suppression of primary antibody responses in Fcgamma receptor-deficient mice. Proc. Natl. Acad. Sci. USA.

[36] Karlsson M.C., Getahun A., Heyman B. (2001). FcgammaRIIB in IgG-mediated suppression of antibody responses: Different impact in vivo and in vitro. J. Immunol..

[37] Kim D., Huey D., Oglesbee M., Niewiesk S. (2011). Insights into the regulatory mechanism controlling the inhibition of vaccine-induced seroconversion by maternal antibodies. Blood..

[38] Niewiesk S. (2014). Maternal antibodies: Clinical significance, mechanism of interference with immune responses, and possible vaccination strategies. Front. Immunol..

